# Time Course Transcriptome Changes in *Shewanella algae* in Response to Salt Stress

**DOI:** 10.1371/journal.pone.0096001

**Published:** 2014-05-01

**Authors:** Xiuping Fu, Duochun Wang, Xiling Yin, Pengcheng Du, Biao Kan

**Affiliations:** 1 State Key Laboratory for Infectious Disease Prevention and Control, National Institute for Communicable Disease Control and Prevention, Chinese Center for Disease Control and Prevention, Beijing, China; 2 Collaborative Innovation Center for Diagnosis and Treatment of Infectious Diseases, The University of Tokyo, Bunkyo-ku, Tokyo, Japan; 3 Department of Cell Biology and Anatomy, Graduate School of Medicine, The University of Tokyo, Bunkyo-ku, Tokyo, Japan; University Paris South, France

## Abstract

*Shewanella algae*, which produces tetrodotoxin and exists in various seafoods, can cause human diseases, such as spondylodiscitis and bloody diarrhea. In the present study, we focused on the temporal, dynamic process in salt-stressed *S. algae* by monitoring the gene transcript levels at different time points after high salt exposure. Transcript changes in amino acid metabolism, carbohydrate metabolism, energy metabolism, membrane transport, regulatory functions, and cellular signaling were found to be important for the high salt response in *S. alga*e. The most common strategies used by bacteria to survive and grow in high salt environments, such as Na^+^ efflux, K^+^ uptake, glutamate transport and biosynthesis, and the accumulation of compatible solutes, were also observed in *S. algae.* In particular, genes involved in peptidoglycan biosynthesis and DNA repair were highly and steadily up-regulated, accompanied by rapid and instantaneous enhancement of the transcription of large- and small-ribosome subunits, which suggested that the structural changes in the cell wall and some stressful responses occurred in *S. algae*. Furthermore, the transcription of genes involved in the tricarboxylic acid (TCA) cycle and the glycolytic pathway was decreased, whereas the transcription of genes involved in anaerobic respiration was increased. These results, demonstrating the multi-pathway reactions of *S. algae* in response to salt stress, increase our understanding of the microbial stress response mechanisms.

## Introduction

*Shewanella* belongs to the order Alteromonadales and the family Alteromonadceae, the latter of which is a member of the gamma subdivision of Proteobacteria [1]. It is a Gram-negative bacterium that is capable of both aerobic and anaerobic respiration. More than 50 species of *Shewanella* have been recognized [2]. *Shewanella* is able to survive in a wide range of environments, including spoiled food and deep-sea and freshwater lake sediment as well as animals’ and patients’ blood and intestines [3]–[11]. Therefore, *Shewanella* has been suggested to be a good candidate model for studying how microorganisms respond to environmental stresses, such as osmolarity, temperature, and pH [12]–[16].

*Shewanella* has been detected in environments ranging from fresh water to hypersaline environments, and it has demonstrated its tolerance to a wide range of salt concentrations. Many *Shewanella* species are marine microorganisms and, therefore, are naturally tolerant to relatively high levels of salt. Common mechanisms that bacteria used to respond to high salinity include the exclusion of harmful ions via a variety of transport systems and the accumulation of compatible solutes through uptake or biosynthesis [17]. For *Shewanella*, a recent study of *S. oneidensis* in elevated salt conditions suggested that the down-regulation of flagellar-related genes might be necessary to conserve energy for sodium transport [18].

*S. algae*, a member of the *Shewanella* genus, can cause human diseases, such as spondylodiscitis and bloody diarrhea [19]–[21]; therefore, it has attracted much attention in microbiology. *S. algae* strains that produce tetrodotoxin and exist in various seafood are recently obtained from anal swabs of patients with food poisoning [22], [23], and the strains are able to grow in high salt levels. The ability to adapt to high salt can be applied to their survival in seafood and other foods that contain high salt levels. In this study, we explored the responses and the possible adaptive mechanisms of *S. algae* strain to elevated salt stress by analyzing the transcriptome profiles of high salt cultures at different time points.

## Materials and Methods

### The Strain

*S. algae* strain 2736 (named MAS2736 previously) was one of strains isolated from the anal swab sample of a food poisoning patient with diarrhea and weak nerval symptom [22], [23]. It can produce tetrodotoxin.

### Growth Conditions

To identify the highest salt concentration that *S. algae* could tolerate, strain 2736 was cultivated for 5 h to an OD_600_ of 1.6 and was then used for seed cultures. The seed cultures were diluted to 1∶100 and then cultivated in triplicate in LB broth (1% tryptone, 0.5% yeast extract) containing 0.5%, 3%, 6%, 8%, or 9% NaCl at 37°C and 200 rpm. The growth rates were measured spectrophotometrically (OD_600_) once every hour in triplicate.

### Sample Preparation

*S. algae* strain 2736 was cultivated in biological triplicate in LB broth (1% tryptone, 0.5% yeast extract, 0.5% NaCl) for 5 h until it reached mid-log phase (OD_600_ = 1.6). Some of the cultures were fixed with a 2∶1 volume of RNA protect Bacteria (Qiagen), harvested by centrifugation (5,500 rpm at 4°C for 10 min), and then were stored at −80°C. The time of culture harvest was defined as time zero. The remaining cultures were harvested by centrifugation (5,500 rpm at 4°C for 10 min) and were resuspended in PBS twice; then, they were cultivated in LB broth containing 8% NaCl. The samples were harvested at four time points (0, 1, 4, and 14 h) in biological triplicate.

### Total RNA Extraction

Total RNA was isolated from the pellet using the RNeasy Mini kit (Qiagen). Genomic DNA was removed by incubation with DNAse (Promega). RNA quality was determined using an Agilent 2100 Bioanalyzer. The extracted total RNA was sequenced.

### RNA Sequencing

rRNA was removed using a kit (BGI Tech) after the total RNA was collected from the prokaryote. Fragmentation buffer was added to disrupt the mRNA into short fragments. Using these short fragments as templates, random hexamer primers were used to synthesize the first-strand cDNA. Second-strand cDNA was synthesized using buffer, dATPs, dGTPs, dCTPs, dUTPs, RNase H, and DNA polymerase I after removing the dNTPs. Short fragments were purified using the QiaQuick PCR extraction kit and were resolved in EB buffer; the ends were repaired, and a poly(A) tail was added. Then, the short fragments were connected to sequencing adapters. The UNG enzyme(BGI Tech) was used to degrade the second-strand cDNA, and the product was purified using the MiniElute PCR Purification Kit prior to PCR amplification. Finally, the library was sequenced using an Illumina HiSeq2000 system.

### Real-time Quantitative PCR (qRT-PCR)

Superscript III first-strand synthesis system (Invitrogen) was used to generate cDNA using 1 µg of RNA and oligo dT primer, according to the manufacturer’s instructions. The qRT-PCR amplifications were performed using the SYBR Green EX Taq mix (TaKaRa) on a Bio-Rad CFX96 Real-Time PCR system. The relative expression level of the specific genes were determined by calculating 2^−ΔΔcq^ compared with the expression level of 0 h time point using 16s rRNA gene as an internal control. The qRT-PCR reactions were performed in triplicate for two biological replications. The specific primers for each gene see Table S1.

Standard curves were generated for each gene to evaluate primer efficiency and for data analysis. *S. algae* strain 2736 was cultivated in biological triplicate in LB broth (1% tryptone, 0.5% yeast extract, 0.5% NaCl) for 5 h (OD_600_ = 1.6) then total RNA was extracted, digested and converted to cDNA. Using 10-fold serial dilutions of the cDNA templates, qRT-PCR amplifications were performed. The standard curve was generated by plotting the Ct values versus ten times serial dilutions of the RNA templates ranging from 2.5×10^0^–2.5×10^−4 ^ng/µl.

### Data Analysis

In our analysis, the expression of each gene at 1 h, 4 h and 14 h was compared with of 0 h, respectively. RPKM (Reads Per kb per Million reads) method [42] was used for the calculation of gene expression difference. False Discovery Rate (FDR) control is used in multiple hypothesis testing to correct for p-value [42]. The genes with FDR ≤0.001 and the ratio greater than 2 were defined as differentially expressed genes (DEGS) compared with 0 h. These DEGS were then used for GO term functional analysis and KEGG pathway analysis.

## Results and Discussion

### Growth Curves of *S. algae* Strain 2736 Under Different Salt Concentrations

**Figure 1:**
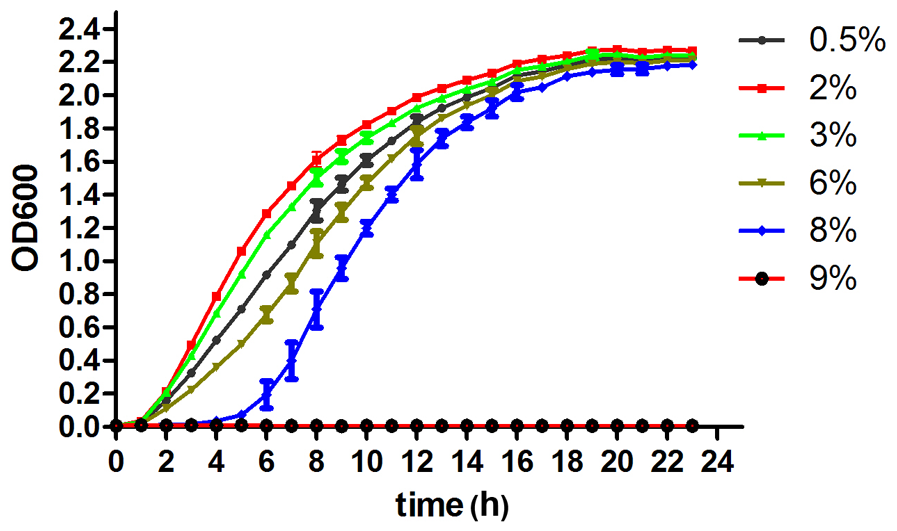
Growth curves for *S. algae* 2736 grown in LB broth. *S. algae* 2736 cells were cultured in LB broth containing 0.5%, 3%, 6%, 8%, or 9% NaCl.

To investigate the concentrations of salt that could sustain the growth of *S. algae*, strain 2736 was cultivated in triplicate in LB broth containing different concentrations of NaCl, ranging from 0.5% to 9%. The growth curves indicated that the optimal growth of strain 2736 is obtained in the presence of 2 to 4% however it is able to grow in presence of 0.5 to 8%. A lag is observed as well as an increased generation time when 8% of NaCl is present in the growth medium. Interestingly, a tremendous decrease in growth was observed between the 8% and 9% NaCl concentrations. Strain 2736 barely grew in LB broth containing 9% NaCl (Fig. 1), suggesting a switch mechanism in the regulation of growth. Under conditions of 8% NaCl, the bacterial growth slowed at the beginning but did reach the stationary phase, similar to growth in lower NaCl concentrations (Fig. 1); therefore, this concentration was regarded as the turning point of the NaCl concentration for the growth of *S. algae* 2736. In our study, 8% NaCl was used for salt stress of the *S. algae* strain 2736.

### Global Transcriptional Changes of *S. algae* 2736 in Response to Salt Stress

We harvested *S. algae* strain 2736 cells exposed to salt stress, and the gene transcript levels were serially monitored at 0, 1, 4, and 14 h. The overall transcriptional profiles revealed that a considerable subset of genes was involved in the response of *S. algae* 2736 to salt stress. To validate the transcriptome data, five open reading frames (ORFs) for the four time points were selected for real-time quantitative PCR analysis. The relative transcript levels were normalized to the levels of 16S rRNA. The results showed that the transcriptome data were highly correlated with the qRT-PCR data (R^2^ = 0.946/0.976/0.934) (Fig. S1). Standard curves were generated for each gene to evaluate primer efficiency and for data analysis. The observed linearity was good for the standard curve over a wide range of cDNA dilutions in triplicate tests (Fig. S2).

**Figure 2:**
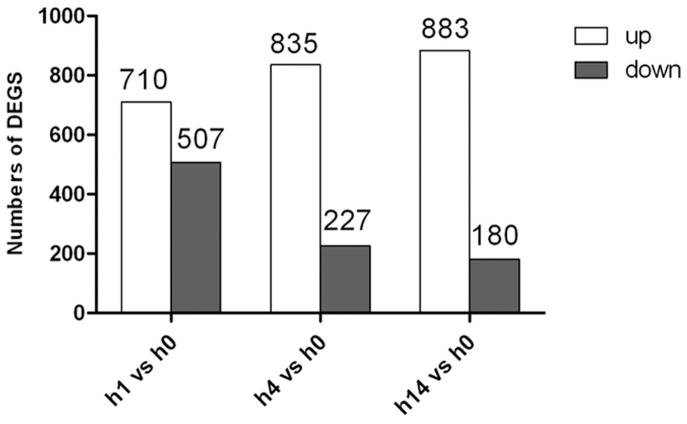
Statistical chart of *S. algae* 2736 DEGS in response to salt stress. Compared to the transcriptional level at 0-regulated and down-regulated, respectively, at 1 h. At 4 h, 835 genes and 227 genes were up-regulated and down-regulated, respectively, whereas 883 genes were up-regulated and 180 genes were down-regulated at 14 h.

We determined that a total of 1808 genes were regulated by salt stress, which represented approximately 41.5% (1808/4354) of the ORFs in the genome of *S. algae* strain 2736 (Table S2). We sequenced the strain 2736 genome using the Illumina method and obtained 4354 predicted CDSs. The genome sequence of 2736 has submitted to GenBank, with the accession number of SRP039467. Compared to the transcriptional level at 0 h, 710 genes and 507 genes were up-regulated and down-regulated, respectively, after 1 h of salt stress. After 4 h of salt stress, 835 genes and 227 genes were up-regulated and down-regulated, respectively. Additionally, after 14 h of salt stress, 883 genes were up-regulated, whereas 180 genes were down-regulated (Fig. 2). At each time point, we identified a larger proportion of up-regulated genes than down-regulated genes, indicating that *S. algae* 2736 cells resisted high salt stress mainly through the up-regulation of genes.

Based on the KEGG pathway analysis, the significantly changed genes were assigned to 19 functional classes. Almost 40.3% of the differentially expressed genes (DEGS) encoded hypothetical proteins, conserved hypothetical proteins, and proteins with unknown functions (499 ORFs or 27.6% of the genes assigned to these categories). The other DEGS were involved in cellular processes and signaling (9.7%), membrane transport (6.0%), carbohydrate metabolism (5.6%), and amino acid metabolism (5.1%), among others (Table 1). Together, almost 20.9% of the genes with significantly altered expression were involved in various metabolic processes. These data indicated that groups of genes involved in various metabolisms, membrane transport, replication and repair, signal transduction, transcription, cell motility, cellular processes and signaling, and translation could play important roles in modulating the cellular activities that allowed *S. algae* 2736 to adapt to salt stress.

**Table 1:** Time course distribution of up- and down- regulated genes within KEGG pathway.

KEGG class2	Total	1 h up(down)	4 h up(down)	14 h up(down)
Amino acid metabolism	93	51(17)	50(2)	33(3)
Carbohydrate metabolism	102	30(48)	36(14)	38(17)
Energy metabolism	69	32(20)	27(7)	40(6)
Lipid metabolism	28	10(2)	18(0)	16(4)
Nucleotide metabolism	32	14(6)	16(6)	9(8)
Glycan biosynthesis and metabolism	11	11(0)	8(0)	6(0)
Metabolism of cofactors and vitamins	34	64(51)	74(23)	76(21)
Xenobiotics biodegradation and metabolism	8	3(4)	3(2)	6(2)
Enzyme families	43	15(11)	22(3)	29(3)
Folding, sorting and degradation	32	15(8)	14(3)	9(5)
Genetic Information processing	68	28(21)	31(7)	29(6)
Cell growth and death	6	2(1)	2(0)	5(0)
Membrane transport	108	30(35)	53(17)	65(11)
Replication and repair	51	26(8)	35(5)	20(0)
Signal transduction	79	24(29)	30(13)	44(8)
Transcription	66	32(19)	30(12)	29(8)
Cell motility	14	4(2)	7(0)	9(0)
Cellular processes and signaling	172	78(43)	90(27)	98(15)
Translation	48	35(1)	10(5)	2(11)
Function unknown	499	177(150)	236(68)	268(42)
NA CLASS	230	78(68)	99(28)	106(29)

**Figure 3:**
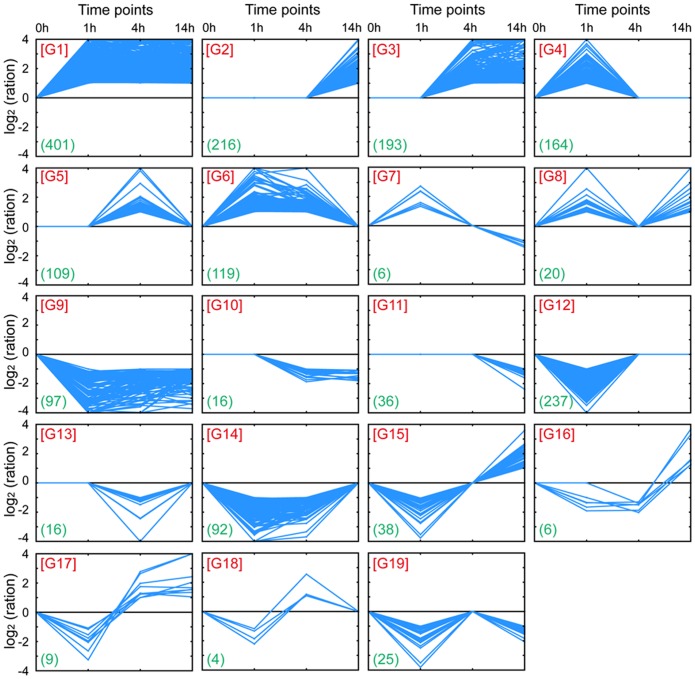
Classification of differentially expressed genes based on the dynamics of the transcript changes during the time course. The number of genes included within each cluster is reported in blue between parentheses.

According to the dynamic changes, the transcriptional profiles were split into 19 clusters (Fig. 3). Clusters 1, 2, and 3 showed a stable up-regulation profile. Cluster 1 showed rapid up-regulation within 1 h, after which the gene profiles obtained stable up-regulation. However, clusters 2 and 3 reached stable up-regulation after a delay of 4 h or 14 h, respectively. Clusters 4, 5, 6, and 7 showed initial up-regulation, but transcriptional levels were restored to the 0 h levels or were down-regulated at the subsequent time points. Cluster 8 showed up-regulation after 1 h, followed by a decrease in transcription levels to the 0 h level at 4 h and a subsequent increase in transcription levels at 14 h. Clusters 9, 10, and 11 reached stable down-regulation at 1, 4, and 14 h, respectively. Clusters 11, 12, 13, 14, 15, 16, 17, and 18 showed initial down-regulation, but transcriptional levels were restored to the 0 h levels or were up-regulated at the subsequent time points. Cluster 19 showed down-regulation after 1 h, followed by an increase in transcription levels to the 0 h level at 4 h and a subsequent decrease in transcription levels at 14 h.

In summary, *S. algae* 2736 cells displayed a wide range of transcriptional alterations after exposure to elevated salt conditions, suggesting that *S. algae* resisted high salt stress through multiple strategies.

### Alterations of High Salt-related Genes in *S. algae* 2736

#### Sigma factor

**Figure 4:**
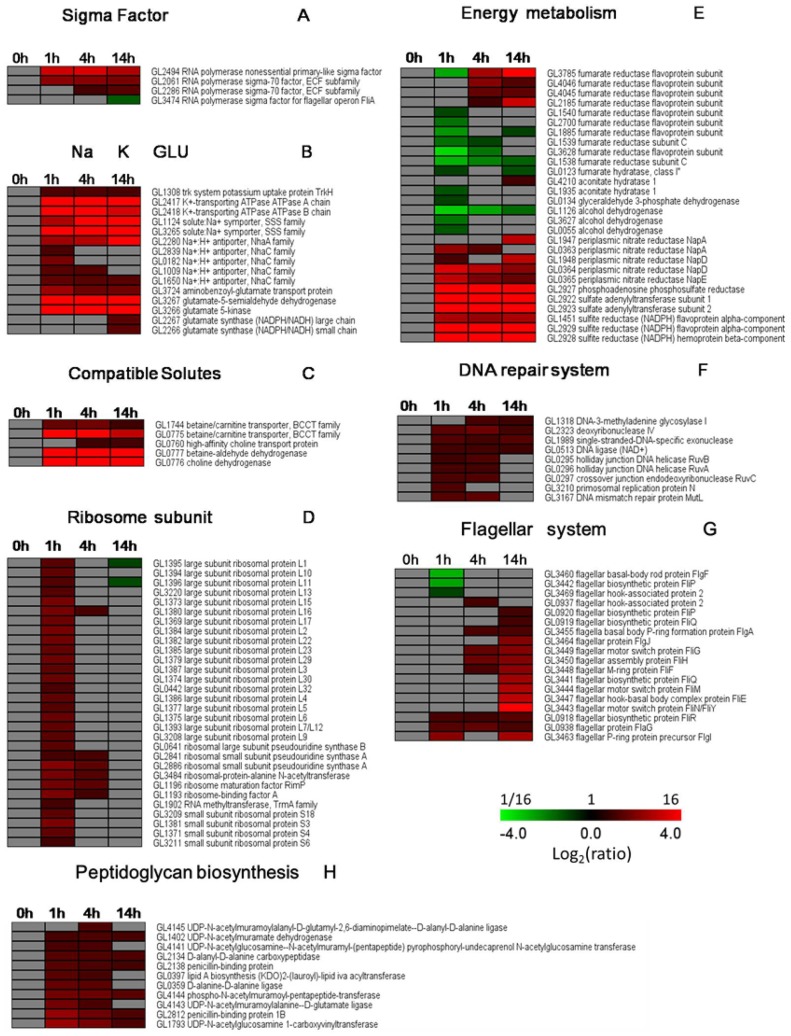
Alterations in high salt-related genes in *S. algae* 2736. The clusters were created using TMEW. (A) Sigma factor. (B) Na+ efflux, K+ uptake, and glutamate accumulation. (C) Accumulation of compatible solutes. (D) Large- and small-ribosome subunits. (E) Energy metabolism. (F) DNA repair system. (G) Flagellar system. (H) Peptidoglycan biosynthesis. Specific colors represent the different regulation patterns. Gray, no change. Red, up-regulation. Green, down-regulation.

In our study, four putative sigma factors were found to be regulated in *S. algae* 2736 after exposure to elevated NaCl conditions, including GL2494, GL2061, GL2286, and GL3474 (Fig. 4A). GL2494, or the RNA polymerase nonessential primary-like sigma factor, which has 75% homology with RpoS, showed significantly sustained up-regulation beginning 1 h after salt stress. RpoS is a global regulatory factor that regulates genes that are mostly related to stress resistance. In *E. coli,* RpoS positively regulates approximately 10% of the genes in the whole genome when the bacterium encounters acute environmental stress, such as hyperosmosis, nutrient deficiency, low pH, and heat shock [24], [25]. GL2061 and GL0228 are RNA polymerase sigma-70 factors that belong to the extracytoplasmic function (ECF) subfamily. ECF sigma factors play key roles during various stress responses and morphological development [26]. GL2061 was immediately up-regulated after salt stress, and it remained at a high level, whereas GL0228 displayed relatively delayed up-regulation, starting 4 h after salt exposure. GL3474, the RNA polymerase sigma factor for flagellar operon FliA, was highly down-regulated at 14 h after salt stress. Our data indicate that sigma factors contributed to the regulation of salt-inducible genes in *S. algae* 2736.

#### Na^+^ efflux, K^+^ uptake, and glutamate accumulation

In *E. coli*, Na^+^ efflux commonly occurs upon exposure to high salt conditions, along with simultaneous activation of K^+^ uptake, thus resulting in high levels of K^+^ ions. Additionally, the cells accumulate glutamate to neutralize the large amounts of cation accumulation [27]. The primary response of *S. algae* 2736 to salt stress is similar to that of *E. coli*. As expected, two genes encoding Na^+^ efflux transporters and five genes encoding Na^+^/H^+^ antiporters were found to be highly up-regulated after 1 h of salt exposure (Fig. 4B). Bacterial cells have three diverse K^+^ transporter systems that maintain the desired concentration of internal K^+^: Kup, Trk, and Kdp [28], [29]. In *S. algae* 2736, *trkH*, *KdpA,* and *KdpB* were significantly up-regulated at all three time points after salt stress (Fig. 4B), demonstrating their importance in the adaptation of *S. algae* to high salt. Additionally, genes encoding the aminobenzoyl-glutamate transporter showed dramatic and immediate up-regulation, especially after 1 h of salt stress. In contrast, the genes encoding the large and small chains of glutamate synthase, which are required for glutamate synthesis, displayed delayed up-regulation patterns and were up-regulated at 14 h after salt stress (Fig. 4B). These data indicate that, at an early stage, the importation of glutamate from the outside was the primary mechanism used to counter high salt stress in *S. algae* 2736. As a secondary response, glutamate synthesis was then activated, resulting in the continual adaption of *S. algae* to its high salinity environment. In contrast, we detected a dramatic and sustained increase in the transcription of genes encoding glutamate-5-semialdehyde dehydrogenase and glutamate 5-kinase (Fig. 4B), two critical enzymes needed to convert glutamate into proline [30]. Taking these data together, we conclude that the genes encoding K^+^ uptake proteins and Na^+^ efflux system components, as well as genes involved in glutamate and proline biosynthesis, were highly induced by NaCl stress in *S. algae* 2736.

#### Accumulation of compatible solutes

The most common strategy used by bacteria to survive and grow in high salt environments is the accumulation of compatible solutes, either by uptake or by biosynthesis, including glycine betaine, choline, carnitine, and trehalose [31]. In *S. algae* 2736, significant changes in compatible solutes were observed at the transcript level under high salt conditions (Fig. 4C). In particular, the expression of the glycine betaine/carnitine transporter was up-regulated at 1, 4, and 14 h after salt stress. The transcript level of the choline transporter was up-regulated beginning at 4 h after salt stress. Two putative genes for betaine synthesis, choline dehydrogenase (BetA) and glycine betaine aldehyde dehydrogenase (BetB), were significantly up-regulated at all three time points in the presence of high salt. These data indicate that *S. algae* 2736 first used betaine as an important compatible solute to protect against the high salt stress, whereas choline played a later role.

#### Large- and small-ribosome subunits

In the present study, we found that the transcript levels of the large- and small-ribosome subunits were significantly enhanced at 1 h after salt stress. However, the levels of most of the large- and small-ribosome subunits were restored to their initial levels at 4 h after salt stress (Fig. 4D), suggesting that *S. algae* entered an adaptive phase. Consistent with our results, a previous study suggested that the removal of a putative ribosome maturation factor conferred salt tolerance on *E. coli* cells [32]. Moreover, the majority of the 70S ribosome was dissociated into subunits after the addition of high concentrations of NaCl, and the dissociated subunits began to reassociate into the 70S ribosome after 4 or 6 h of salt stress. It has been suggested that the ribosome mediates a novel stress response pathway [33]. Taken together, the large- and small-ribosome subunits appeared to play roles in the adaption of *S. algae* to high salt stress.

#### Energy metabolism

It has been suggested that critical enzymes involved in both aerobic and anaerobic respiration were significantly up-regulated in salt-stressed bacterial cells [18], [34]–[37]. Notably, in our study, the transcript levels of fumarate reductase and aconitate hydratase, two critical enzymes necessary for the tricarboxylic acid (TCA) cycle, were down-regulated in *S. algae* 2736 cells after 1 h of salt stress, and these levels increased again to the 0 h level or further increased at 4 h and 14 h. Similarly, glyceraldehyde 3-phosphate dehydrogenase and alcohol dehydrogenase, key enzymes involved in the glycolytic pathway, were down-regulated at the transcriptional level at 1 h after salt stress and returned to the 0 h level after 4 h of salt stress. However, enzymes critical for anaerobic respiration, such as periplasmic nitrate reductase, sulfite reductase, and sulfate reductase, were significantly enhanced at the transcriptional level in *S. algae* 2736 cells at the three time points after high salt exposure (Fig. 4E). Based on these findings together, we conclude that, during the initial stage after exposure to high salt concentrations, *S. algae* 2736 appears to utilize anaerobic respiration for energy production, instead of the tricarboxylic acid (TCA) cycle and the glycolytic pathway.

#### DNA repair system

The overall transcriptome profiles demonstrated that genes involved in DNA base excision repair, mismatch repair, and homologous recombination were dramatically up-regulated in the salt-stressed *S. algae* 2736 cells, especially at 1 h and 4 h after salt exposure (Fig. 4F). Three DNA helicases encoded by RuvABC were up-regulated at 1 h and 4 h after salt stress. RuvB might function at the Holliday junctions to overcome regions of DNA heterology and DNA lesions [38]. DNA helicases are known confer high salinity tolerance in tobacco [39]. These results indicate that salt stress might induce the impairment of nucleic acid synthesis, thereby triggering the corresponding repair systems.

#### Peptidoglycan biosynthesis

We observed that key genes related to peptidoglycan biosynthesis were up-regulated in *S. algae* 2736 cells by salt stress (Fig. 4H). It has been documented that several physical changes, such as dehydration and shrinkage of the cells, occur immediately in *E. coli* cells after osmotic shock caused by an increase in salt concentration, in response to the changes in environmental osmolarity [40], [41]. Therefore, our data also indicate that peptidoglycan, the dominant component of the Gram-negative bacterial cell wall, serves as an osmoprotectant and, therefore, helps *S. algae* to combat the dehydration caused by high levels of salt.

#### Flagellar system

Among all of the flagellar-related genes, three genes were down-regulated, whereas another three genes were up-regulated at 1 h after salt exposure. Importantly, seven genes were up-regulated at 4 h after high salt exposure, and 14 genes were up-regulated at 14 h after salt administration. No genes were found to be down-regulated at either 4 h or 14 h after salt stress (Fig. 4G). Previous studies have documented in detail the transcriptional regulation of flagellar genes in bacterial species. For example, *S. oneidensis* MR-1 responded to elevated salt concentrations by down-regulating flagellar assembly genes, accompanied by a decrease in cell motility. A dynamic process was observed in *Desulfovibrio vulgaris* Hildenborough. During early time points during salt stress, many chemotaxis-related genes were found to be up-regulated; at later time points during salt stress, only a few such genes were overexpressed, and the expression of most genes remained unchanged [18], [34]–[36]. Our results were not consistent with the findings above, and one possible explanation could be that the up-regulation of flagellar genes at 14 h might not be related to the response to high salt but was instead related to the growth state of the bacteria.

In conclusion, in this study, we explored the possible mechanisms of the adaptation of *S. algae* to high salt conditions by performing transcriptome profiling combined with cell growth analyses. A broad set of differentially expressed genes was observed. Globally, genome-wide transcriptional analyses demonstrated that transcript changes, especially changes in amino acid metabolism, carbohydrate metabolism, energy metabolism, membrane transport, regulatory functions, and cellular signaling, seemed to be important for *S. algae* to respond to salt stress.

**Figure 5:**
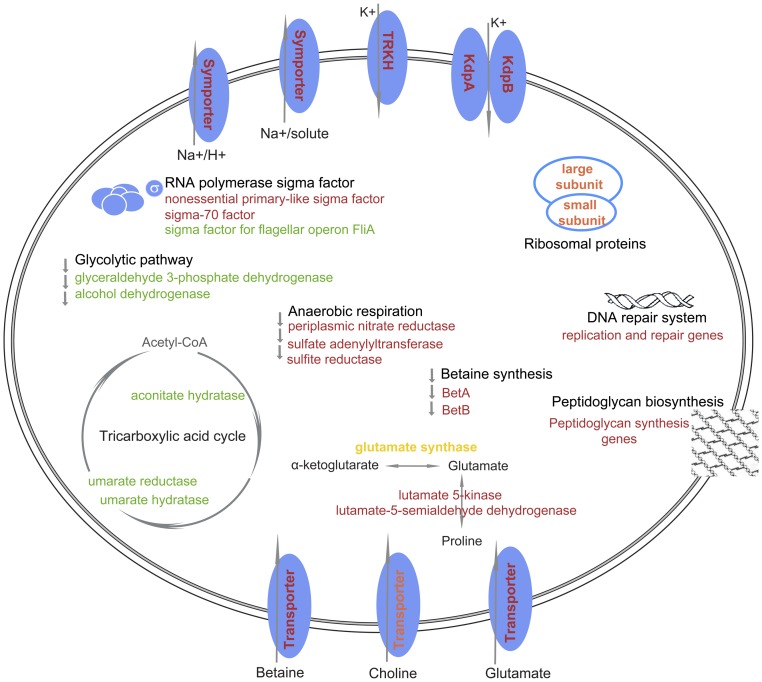
Conceptual model of *S. algae* responses to the exposure to high NaCl concentration. The protein elements involved in the changed pathways were showed and marked with different colors: Red indicates the transcription increase at all three time points (1 h, 4 h, 14 h); Orange indicates the increase at 1 h or 4 h, or both 1 h and 4 h; Yellow indicates the increase at 14 h; Green indicates the decreased transcription at any one time point, no increase was observed in all three time points. The names of the pathways and the transported substrates were shown as black fonts.

Importantly, we elucidated a temporal, dynamic process in the salt-stressed *S. algae* strain 2736 by monitoring gene transcription levels at 0, 1, 4, and 14 h. Exposure of *S. algae* to high salt could induce two different, but related, responses, which we defined here as the initial and prolonged responses. Initially, in response to the high salt environment, the *S. algae* strain quickly recruited central factors, such as K^+^, glutamate, and betaine, mainly through import, as represented by the apparent up-regulation of certain transport genes (e.g., K^+^ transporters, aminobenzoyl-glutamate transports, and glycine betaine/carnitine transporter) at 1 h. Na^+^ efflux, the accumulation of large−/small-ribosome subunits, and the induction of the DNA repair system appear to be other initial mechanisms of the response of *S. algae* to elevated salt stress. It is worth noting that *S. algae* can rely on anaerobic respiration but not the tricarboxylic acid (TCA) cycle or glycolytic pathway for energy production during the initial stage of high salt exposure. When *S. algae* was persistently exposed to high salt concentrations, as a prolonged response, corresponding biosynthesis systems were triggered to play vital roles, resulting in the adaption of *S. algae* to salt stress. For instance, the expression of choline transport protein was increased beginning at 4 h after salt stress. However, some prolonged regulated genes did not appear to be related to the high salt response but were instead related to the growth state of the bacteria. For example, the expression of flagellar genes and glutamate synthase were increased beginning 14 h after high salt exposure (Fig. 5).

Taking all of our findings together, we present a temporal, dynamic response pattern of *S. algae* strain 2736 after exposure to high salt conditions, and our results elucidate the mechanisms that *Shewanella* utilizes to survive and adapt to environmental stress.

## Supporting Information

Figure S1 **Correlation of real-time qRT-PCR and RNA sequencing analyses.**
(TIF)

Figure S2 **The standard curves for each gene.**
(TIF)

Table S1 **qRT-PCR Primers.**
(DOCX)

Table S2 **The raw data and the complete list of up and down regulated genes.**
(XLS)
