# Supplementary figures and images for: Time Course Transcriptome Changes in Shewanella algae in Response to Salt Stress
Source: PLoS One. 2014 May 1;9(5):e96001. doi: 10.1371/journal.pone.0096001 (PMC4006864; doi:10.1371/journal.pone.0096001)

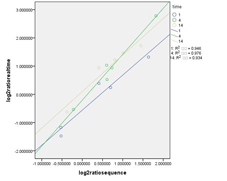

Supplement: Figure S1 — Correlation of real-time qRT-PCR and RNA sequencing analyses. (TIF) [file pone.0096001.s001.tif]

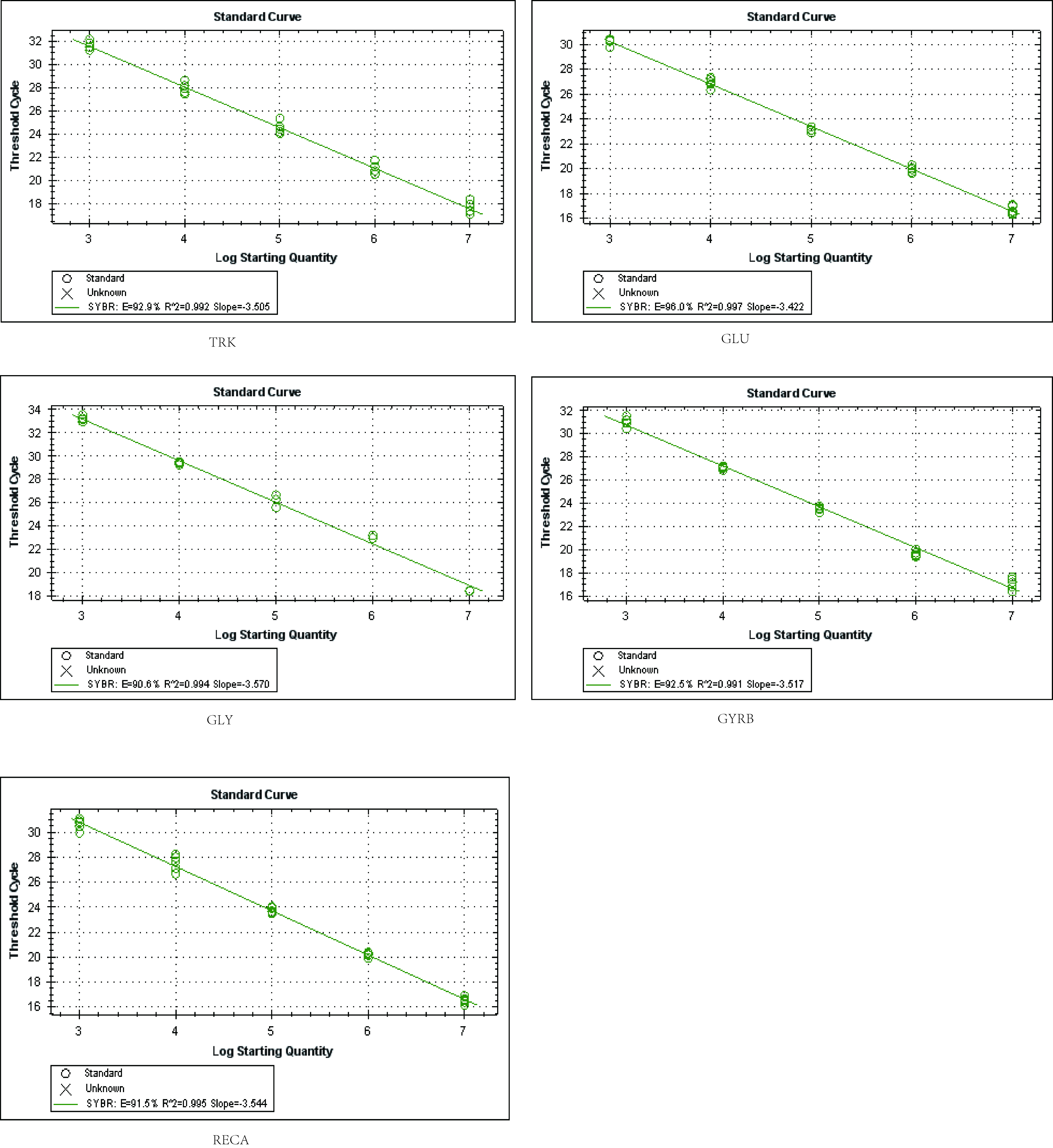

Supplement: Figure S2 — The standard curves for each gene. (TIF) [file pone.0096001.s002.tif]
