# Supplementary material for: Time Course Transcriptome Changes in Shewanella algae in Response to Salt Stress
Source: PLoS One. 2014 May 1;9(5):e96001. doi: 10.1371/journal.pone.0096001 (PMC4006864; doi:10.1371/journal.pone.0096001)
Supplement: Table S1 — qRT-PCR Primers. (DOCX) [file pone.0096001.s003.docx]

Supplement Table S1：qRT-PCR Primers

| Gene id | Function | Prime |
| --- | --- | --- |
| MAS2736GL001744 | betaine/carnitine transporter, BCCT family | TCTTGGGTAACTTCGGCTTGT |
|  |  | CCAGATAGCTGAACGGCAACT |
| MAS2736GL002267 | glutamate synthase (NADPH/NADH) large chain | GCGCTCGCTGTATGACTATTT |
|  |  | GTGTCCCGTGGTTTCGTT |
| MAS2736GL001303 | trk system potassium uptake protein TrkH | CTATGGCCTGACCGACTATGG |
|  |  | GCGATCTGGAAGCGGAATATC |
| MAS2736GL002498 | recombination protein RecA | TGAAGTGGCTGCCGAAATC |
|  |  | TTCTCCGGTTTCCAGATCGAC |
| MAS2736GL001322 | DNA gyrase subunit B | AATGCTGGCCGATGAGG |
|  |  | AATGAGCTGCGGCAGATACAC |
